# Miniature 3D-printed rod-like refractive objective for endoscopic applications

**DOI:** 10.1117/1.JBO.31.3.036003

**Published:** 2026-03-10

**Authors:** Kevin Beckford, Yicheng Ma, Jinyun Liu, Tomasz S. Tkaczyk

**Affiliations:** a Rice University, Department of Electrical and Computer Engineering, Houston, Texas, United States; b Rice University, Department of Bioengineering, Houston, Texas, United States

**Keywords:** miniature endoscopic objectives, fluorescence endoscopy, additive manufacturing, two-photon polymerization

## Abstract

**Significance:**

The three-dimensional (3D)-printed refractive rod objective for endoscopy preserves the compact, gradient index (GRIN)-like format while delivering a wide field of view (FOV) comparable to its diameter and larger than that of a commercial GRIN lens.

**Aim:**

We focus on the design, fabrication, and experimental validation of a proof-of-concept refractive rod objective for fluorescence imaging of mouse colon tissue, with performance compared with a commercial GRIN lens.

**Approach:**

A 1× magnification refractive rod-like objective was designed in Zemax OpticStudio and fabricated using two-photon polymerization additive manufacturing. The objective consists of a sequence of convex refractive surfaces printed in contact at their vertices, with refractive index contrast provided by partially or non-polymerized resin contained within an enclosing wall. The lens has a diameter of 500  μm with a clear aperture of 470  μm, a total length of 2.06 mm, and a working distance of 1.6 mm and was optimized for a numerical aperture of 0.075 and a 500-μm design field at 525-nm light. Three photopolymer resins (IP-S, IP-Visio, and IPX-Clear) were evaluated through excitation–emission matrix measurements to assess autofluorescence. By imaging group 7, element 6 of a 1951 United States Air Force (USAF) resolution target, the field was assessed by plot profile and modulation transfer function measurements.

**Results:**

The fabricated objective resolved group 7, element 6 of a USAF resolution target (4.38  μm), closely matching the theoretical diffraction-limited resolution of 4.27  μm. Compared with a commercial 600-μm-diameter GRIN lens, the 3D-printed objective achieved a substantially larger FOV (498 versus 188  μm). Spectral characterization showed that IP-Visio exhibited the lowest autofluorescence under 455-nm excitation when unpolymerized resin was present. Using an IP-Visio objective, fluorescence images of proflavine-stained mouse colon tissue were successfully acquired.

**Conclusions:**

The demonstrated refractive rod-like objective combines the compact geometry of a GRIN lens with the aberration correction capability of multi-element refractive optics, enabling uniform resolution across a large FOV. The approach also allows material selection tailored to fluorescence imaging requirements. Future work will focus on integration with fiber bundles and on fully polymerized designs with spatially tuned refractive index for improved long-term stability.

## 1 Introduction

One of the optical elements commonly used in many endoscopic applications is the gradient index (GRIN) lenses.^1^^,^^2^ GRIN lenses are compact rod components that use the change of refractive index within the lens (varied perpendicularly to the lens axis). The change in refractive index is primarily achieved through chemical processes.^3^^,^^4^ However, alternative fabrication methods that do not rely on such chemical processes include direct ink writing,^5^ digital light processing, vat photopolymerization,^6^ and two-photon polymerization (2PP) three-dimensional (3D) printing.^7^ Although optical rays are being bent continuously, the length of the GRIN lens relative to its pitch determines its actual function of focusing, collimating, or imaging. The benefit of this lens is that it is compact (typically 0.2 to 2.0 mm in diameter), relatively low-cost, and easily integrated with optical systems.^8^ The principle of gradient change in refractive index results in a degradation of image quality away from the on-axis. At the edge of the field of view (FOV), GRIN lenses provide a lower numerical aperture (NA) relative to the center of the FOV. To correct the performance of GRIN lenses, some have used adaptive optics to increase the resolution off-axis by correcting the aberrations away from the axis.^9^^–^^11^ This results in a larger, costly, and complex optical system. One way to overcome this without increasing the complexity and the footprint of the system is by reverting to the conventional nature of refractive lenses. The advantage of using a traditional refractive lens is that it can be corrected to maintain its performance over the entire FOV. To leverage the compact, rod-like form factor of a GRIN lens while avoiding reliance on a parabolic refractive index profile for focusing, we propose the fabrication of a compact, rod-shaped lens composed of a sequence of convex refractive elements embedded in a lower refractive index material using a 2PP additive manufacturing technique. This results in a solid rod-like component with no need for assembly or its tight tolerance. As the refractive index changes between polymerized and partially/non-polymerized resin is small, the lens requires a sequence of refractive surfaces. The number of these surfaces allows efficient correction for wave aberrations. To contain the unpolymerized resin and ensure mechanical stability, an outer enclosure is printed as part of the lens structure, resulting in a fully enclosed, self-contained rod-like objective. Note that, because the 2PP additive manufacturing technique has sub-micron positioning precision, the lens does not suffer from assembly errors. All surfaces were fabricated in a single process using a 2PP additive manufacturing technique with the Nanoscribe Quantum X system. The calibrated printing FOV zone of the 25× objective in the Quantum X is 700  μm, allowing the objective to be fabricated within a single printing zone without stitching. The printed objective has a diameter of 480 to 500  μm, depending on the resin used for printing. Although miniature objectives have been fabricated for endoscopy applications using 2PP technology^12^^–^^14^, the 3D-printed rod-like objective is a fully enclosed, rod-like form factor component fabricated as a single monolithic print, in which optical power and aberration correction are realized through a sequence of polymerized–unpolymerized refractive transitions within the cylindrical rod. This proof-of-concept architecture preserves a compact GRIN-like mechanical configuration while avoiding multi-element micro-assembly and alignment. Moreover, the enclosed liquid region is inherently less sensitive to printed surface roughness, motivating future iterations that can use larger print heads to reduce fabrication time while maintaining optical performance. Looking forward, this solid–liquid architecture represents a step toward a fully polymerized rod objective, where refractive index variation is achieved by modulating the local degree of polymerization through controlled exposure power—ultimately enabling a rigid, all-solid refractive design with embedded optical functionality.

## 2 Methods and Materials

**Fig. 1:**
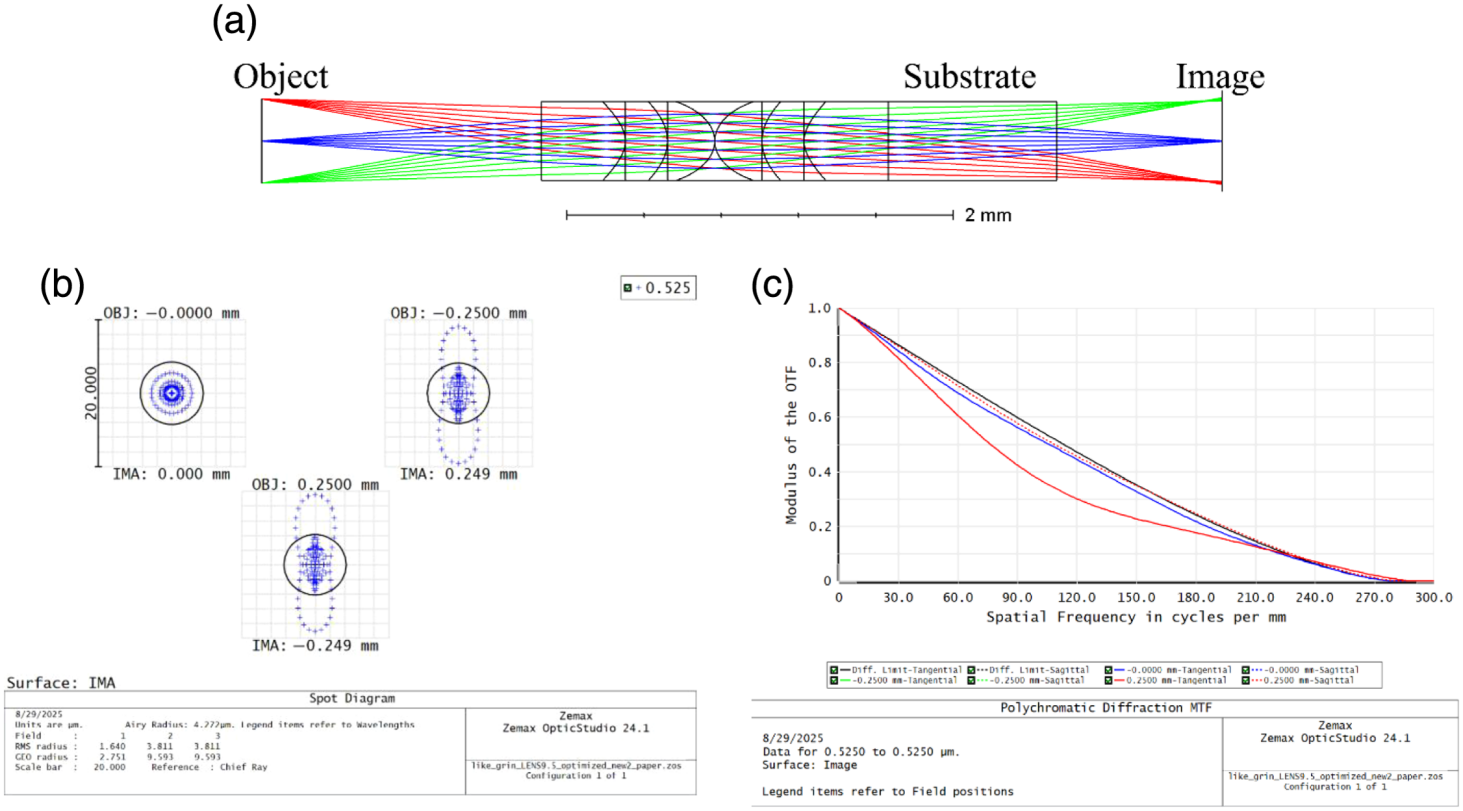
(a) Optical schematic of the 1× objective; the thickness of the substrate it is attached to is added in the design. (b) Spot diagrams at 0-, −0.25-, and 0.25-mm fields. (c) MTF plots include distribution for 0-, −0.25-, and 0.25-mm fields.

The proof-of-concept rod-like lens was designed using OpticStudio (ZEMAX) and optimized to achieve a resolution of ∼4  μm, suitable for imaging cellular features in biological tissues such as the colon. The objective was designed for operation with proflavine and fluorescein isothiocyanate dyes with center emission peaks of 511 and 535 nm, respectively. Therefore, it was optimized for the central wavelength within the 500- to 550-nm spectral range, corresponding to the emission of proflavine. For this proof-of-concept rod-like objective, the clear aperture was set to 470  μm in diameter, whereas the enclosing wall (barrel) thickness ranged from 10 to 30  μm, depending on the polymer used. A 10-μm wall was sufficient for IP-S, whereas both IP-Visio and IPX-Clear required a thicker wall to prevent mechanical instability and bulging of the objective. The image-space NA of 0.075 was selected to meet the targeted resolution range. Most of these design goals are shown in Table 1. The objective was designed with a working distance of 1.6 mm and a total length of 2.06 mm. The substrate thickness was accounted for in the optical model, as the printed lens remained attached to the fused silica substrate for testing purposes. In the future, an objective will be reoptimized for integration with a fiber bundle. The lens elements were placed in direct contact at their vertices to minimize total system length and enhance mechanical stability. The refractive index of the polymerized lens material was defined as 1.515, whereas the liquid resin among lens elements was modeled with a refractive index of 1.486.^15^ The miniature objective was optimized for diffraction-limited performance across a 500-μm FOV. Based on the Rayleigh criterion, the theoretical resolution was calculated to be 4.27  μm at 525 nm. The optical schematic of the rod-like objective along with its simulated airy disk and modulation transfer function (MTF) is shown in Fig. 1.

**Table 1:** Optical design goals.

Image space NA	0.075
Object space NA	0.075
Working distance	1.6 mm
Magnification	1×
Length of rod-like lens	2.06 mm
Clear aperture	470 μm
FOV	500 μm
Wavelength	525 nm

**Fig. 2:**
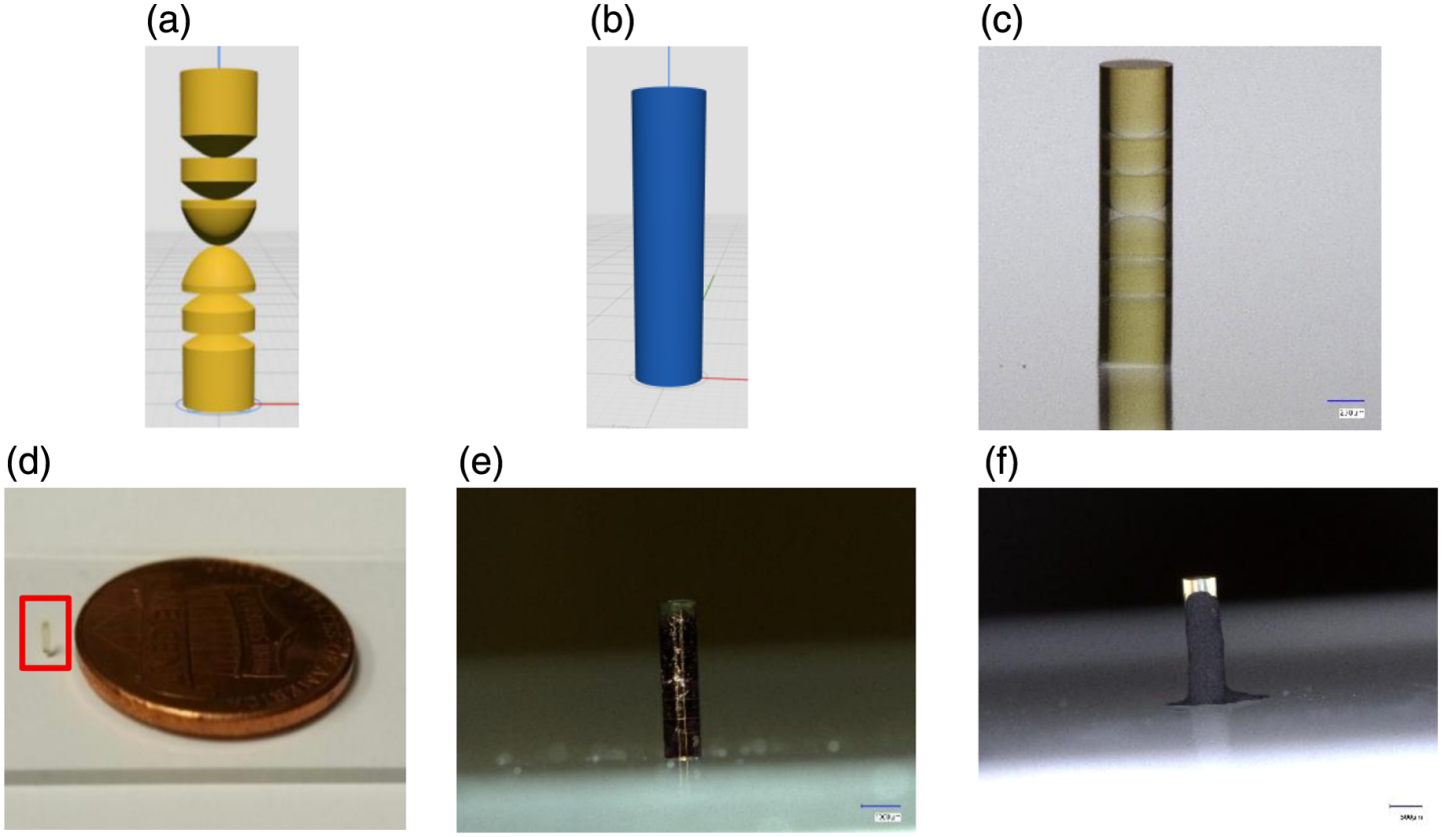
(a) Lenses printed using 2GL mode to ensure smooth optical surfaces. (b) Outer housing wall printed using the fast-print 2PP setting. (c) Microscope image of the completed objective taken with a Keyence system (scale bar: 250  μm). (d) Photograph of the printed objective placed next to a United States penny for size reference. (e) Objective after being coated with black marker to reduce stray light (scale bar: 500  μm). (f) Objective after being coated with Musou black to reduce stray light (scale bar: 500  μm).

After optimization in ZEMAX, the lenses and outer wall were created in NanoprintX as shown in Figs. 2(a) and 2(b). This is Nanoscribe proprietary software that allows for 3D printing with two-photon grayscale lithography (2GL). 2GL printing mode modulates the voxel size by changing the laser power to match the surface shape.^16^ The primary advantage of 2GL is its ability to fabricate structures without compromising surface quality at significantly higher speeds compared with conventional 2PP methods that rely on low slicing and hatching distances that substantially increase the printing time.^17^ 2GL mode is used to fabricate the lenses, whereas the 2PP speed-print setting—which uses high slicing and hatching distances—is employed for printing the outer wall. The objective was printed first with the 25× objective, and the IP-S resin was polymerized. For the 2GL mode, the laser power was set to 70 mW, the scan speed to 200,000  μm/s, with a slicing distance of 1.2  μm and a hatching distance of 0.25  μm. In contrast, the 2PP speed-print preset used a laser power of 100 mW, scan speed of 250,000  μm/s, slicing distance of 2.4  μm, and hatching distance of 0.5  μm. The total print time for the miniature objective lens was 2 h and 39 min. After fabrication, excess unpolymerized resin was removed by immersing the sample in SU-8 developer for 18 min, followed by a 2-min rinse in Isopropyl Alcohol (IPA). Figures 2(c) and 2(d) show the completed miniature objective. To reduce stray light, the perimeter of the objective was painted black using a black Sharpie marker, as shown in Fig. 2(e). Using Fresnel equations and off-axis ray tracing in ZEMAX, the total expected reflectance from internal lens surfaces within the objective is ∼0.2%. The largest contributions to stray light arise from the air–objective interface and the substrate–air interface. In Fig. 2(f), a separate objective was coated with Musou Black (Koyo Orient Japan Co., Ltd., Ageo, Japan), which is formulated for ultra-low reflectance/high absorption in the visible range, cures at room temperature, and is cost-effective; Musou Black has also been evaluated in an optics context for stray-light suppression.^18^ To validate its suitability here, we measured absorption using a TECAN Spark Fusion microplate reader (96-well plate, 500 to 650 nm), where Musou Black maintained ∼0.96 absorption across the measured band and exhibited higher and more uniform absorption than Sharpie with absorption of ∼0.65 on average. Fluorescence was additionally assessed under 444- to 460-nm excitation with 511-nm emission (proflavine peak), and both Musou and Sharpie produced signals comparable to a blank well, indicating negligible coating fluorescence under measurement conditions. The application of a thin, uniform black layer was easier with the Sharpie marker than with the Musou paint, because the denser paint is more difficult to apply evenly. Next, the fabrication procedure was repeated using IP-Visio resin and IPX-Clear. As the refractive indices of IP-Visio and IPX-Clear differ from that of IP-S, each corresponding objective was reoptimized in ZEMAX to maintain diffraction-limited performance. The prescriptions for all objectives designed in IP-S, IP-Visio, and IPX-Clear are provided in Supplementary Material. Although IP-S is recommended due to its higher print fidelity and mechanical stability, it also exhibits autofluorescence when working with short excitation wavelengths. IP-Visio was developed by Nanoscribe to lower autofluorescence from the printed structures. To accommodate material differences, printing parameters such as laser power, scan speed, slicing, and hatching distances were adjusted. IP-Visio requires a higher dosage compared with IP-S, meaning that the scan speed and slicing distance had to be lowered. As a result, a scan speed of 75,000  μm/s and a laser power of 110 mW were applied. The slicing distance was set to 0.5  μm, and the hatching distance was set to 0.3  μm. The 2GL preset was not used since the printing parameters needed to be optimized for this specific resin. These changes in printing parameters resulted in print lengths of ∼9  h. This can be shortened in the future after optimizing/defining 2GL presets for the material. IPX-Clear is a new material that was developed to have the highest transmission out of all the other IP-Resins while having low auto-fluorescence when fully polymerized.^15^ The print parameters used for the fabrication of this material rod-like objective were a scan speed of 200,000  μm/s, laser power of 38 mW, slicing distance of 1.2  μm, and a hatching distance of 0.25  μm. The entire rod-like objective was fabricated in a 2GL preset and printed for a total of 3 h and 17 min.

## 3 Results

**Fig. 3:**
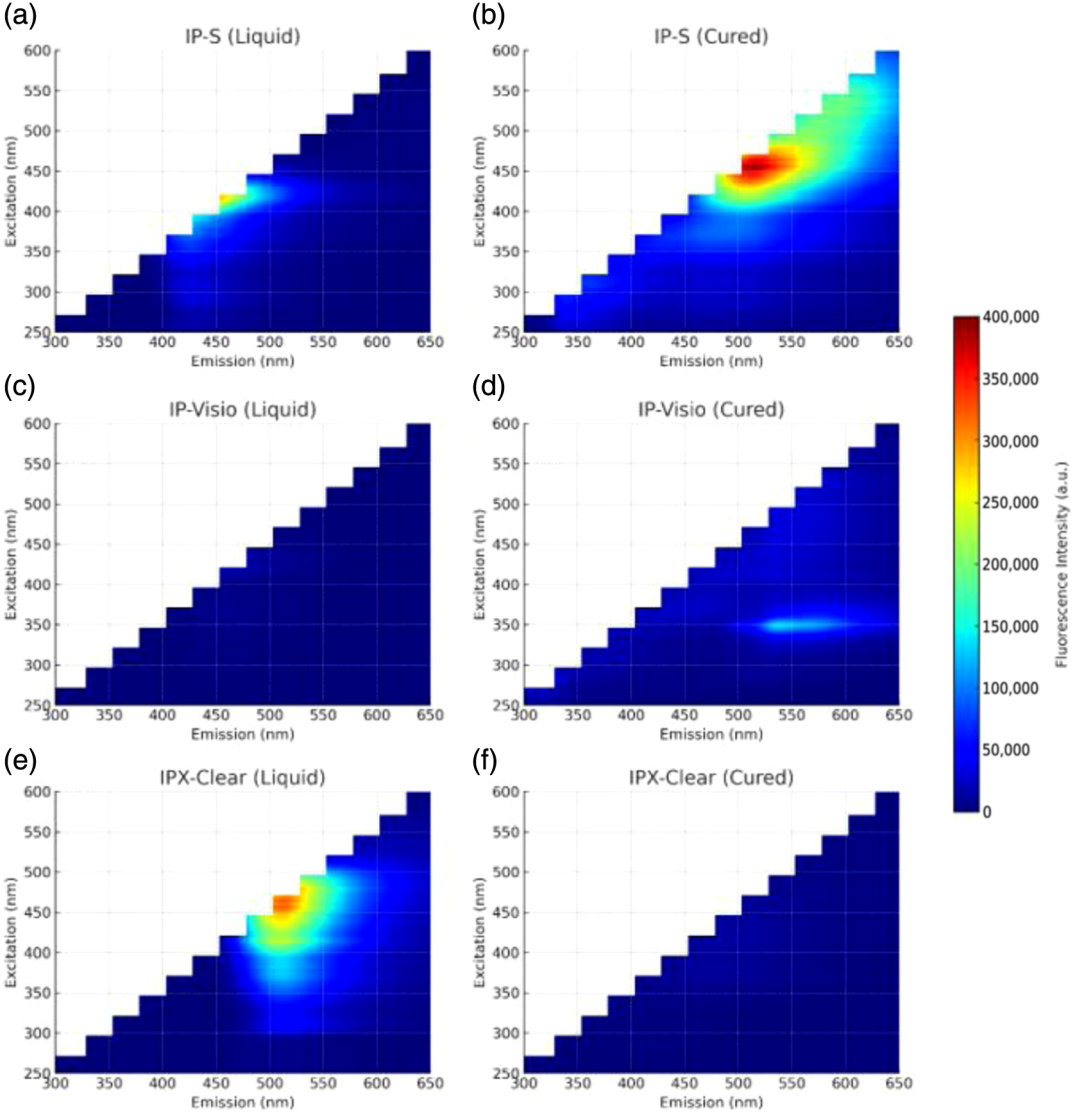
(a) IP-S liquid resin excitation versus emission fluorescence two-dimensional (2D) EEM graph. (b) IP-S-cured resin excitation versus emission fluorescence 2D EEM graph. (c) IP-Visio liquid excitation versus emission fluorescence 2D EEM graph. (d) IP-Visio liquid excitation versus emission fluorescence 2D EEM graph. (e) IPX-Clear liquid excitation versus emission fluorescence 2D EEM graph. (f) IPX-Clear cured excitation versus emission fluorescence 2D EEM graph.

To identify the material with the lowest autofluorescence within the desired excitation range, fluorescence measurements were performed using a TECAN Spark microplate reader. Although autofluorescence for IP-S and IP-Visio has previously been reported at a single excitation wavelength,^19^ here, a full 3D excitation–emission matrix (EEM) was obtained for IP-S, IP-Visio, and IPX-Clear in both liquid and ultraviolet (UV)-cured forms, covering excitation from 250 to 600 nm and emission from 300 to 650 nm. In their liquid states, IP-S and IP-Visio appear colorless, whereas IPX-Clear exhibits a red tint. After UV curing for 60 min, IP-S acquires a green tint, whereas IP-Visio and IPX-Clear remain clear. The results shown in Fig. 3 demonstrate that cured IPX-Clear and liquid IP-Visio exhibit the lowest autofluorescence in the visible spectrum. In contrast, IP-S shows the highest autofluorescence, with the cured form emitting strongly under 430 to 550 nm excitation and the liquid form under 375 to 440 nm excitation. For IP-Visio, the cured form exhibits elevated fluorescence intensity at 325 to 350 nm excitation, whereas the liquid form shows increased fluorescence intensity at 400 to 435 nm excitation. These findings indicate that IP-S is the least suitable for fluorescence imaging of dyes with emission peaks below 600 nm (e.g., proflavine), IPX-Clear cured has the lowest autofluorescence but suffers from high fluorescence in its liquid state, and IP-Visio is the most advantageous, maintaining relatively low autofluorescence in both liquid and cured forms.

**Fig. 4:**
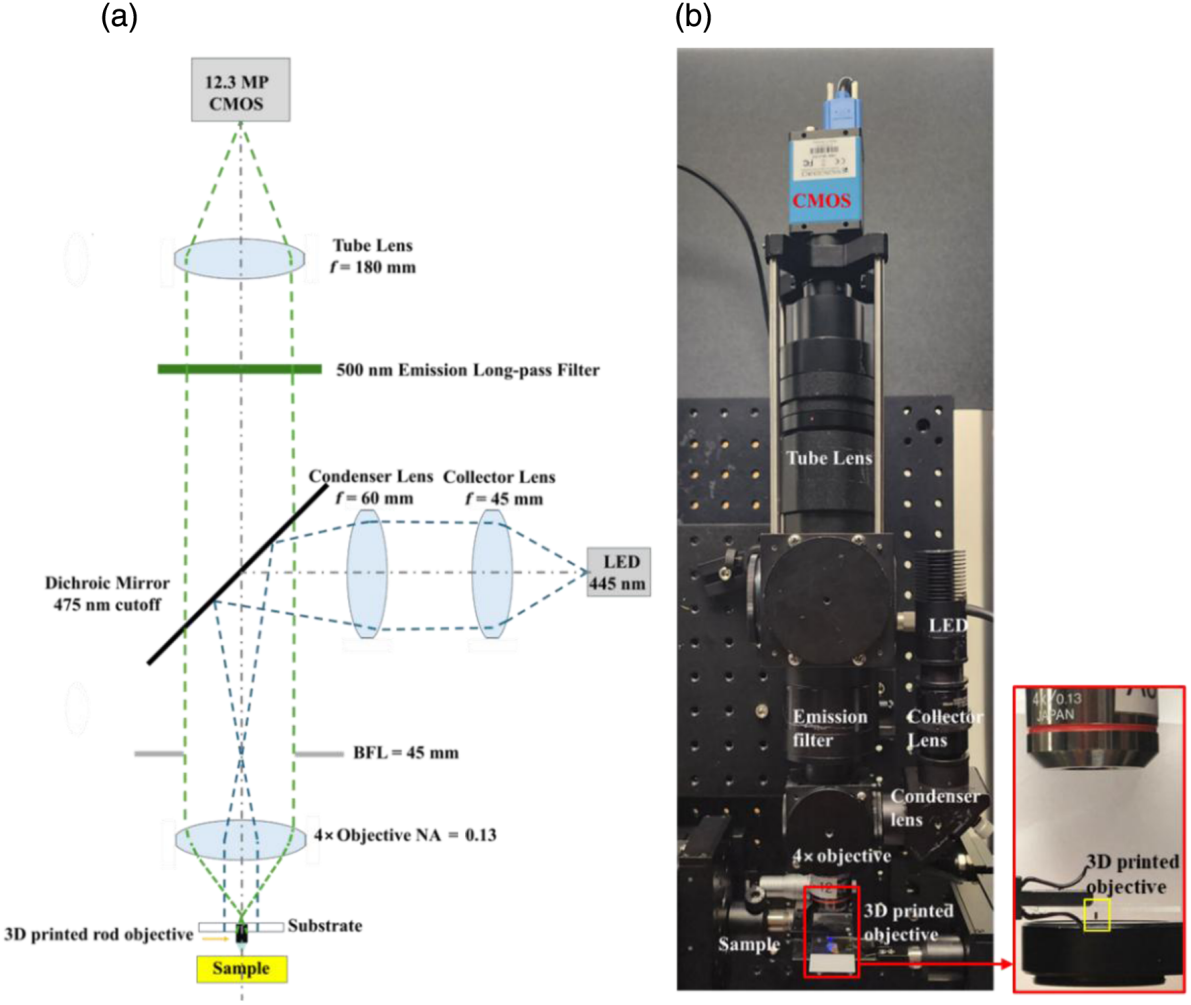
(a) Schematic of the optical system. (b) Annotated picture of the optical setup.

To evaluate the proof-of-concept objective, we used an in-house fluorescence imaging system previously developed for detecting cervical intraepithelial neoplasia^20^ as shown in Fig. 4. Minor modifications were made, including replacing the camera and removing the excitation filter. In this work, the in-contact faceplate from the original system was replaced with either the 3D-printed rod-like objective or a commercial GRIN lens. The rod-like lens was mounted using a standard microscope slide holder, whereas the GRIN lens was secured with a fiber chuck. The imaging system shown in Fig. 4 was used for all imaging tests with both the commercial GRIN lens and the 3D-printed objective. White light transmitted through a green filter (BrightLine, FF03-525/50-25) was used to test the resolution of the 3D-printed rod-like objective. For fluorescence imaging, proflavine dye was used to stain tissue, with an excitation peak at 443 nm and an emission peak at 511 nm. Excitation was provided by a 455-nm light-emitting diode (LED) (M455L2, Thorlabs Inc., Newton, New Jersey, United States), and the resulting emission was directed to a 12.3-MP camera (DMK 38UX253, The Imaging Source, Bremen, Germany) after passing through a 4× 0.13 NA objective (Olympus UPlanFl, Tokyo, Japan), a 475-nm cutoff dichroic mirror, a 500-nm long-pass filter, and finally a compatible tube lens (U-TLU, Olympus).

**Fig. 5:**
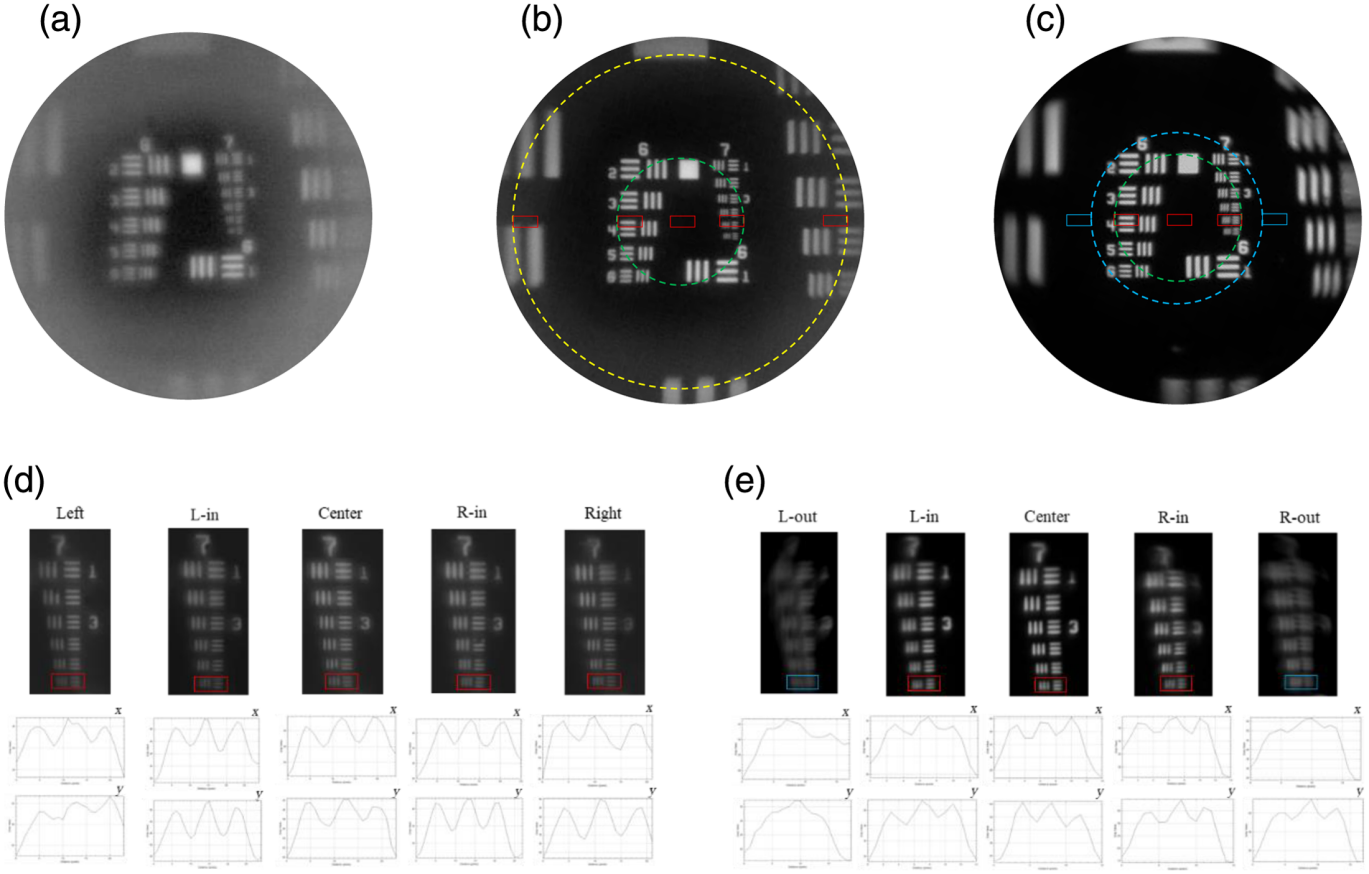
(a)–(c) Target imaged with the (a) uncoated rod-like objective, (b) black-coated rod-like objective, and (c) commercial GRIN lens. (b) and (c) FOVs of the 3D-printed objective and the GRIN lens, respectively, with yellow and green circles indicating the regions where group 7, element 6 is resolvable. The yellow circle has a measured diameter of 498  μm, whereas the green circle measures 188  μm. (b)–(e) Rectangular boxes mark the positions where group 7, element 6 was evaluated. Line profiles were plotted for both the vertical line pairs (x) and horizontal line pairs (y) to confirm whether the element was resolvable. (d) Selected regions of the USAF resolution target at −249  μm (left), −94.5  μm (L-in), 0  μm (center), +94.5  μm (R-in), and +249  μm (right) demonstrate that the dashed, yellow-circled region is resolvable. (e) Regions at −106.5  μm (L-out), −94.5  μm (L-in), 0  μm (center), +94.5  μm (R-in), and +106.5  μm (R-out) show that the dash green-circled field is resolvable, whereas the blue region is not. For the region between the green and blue circles, only one direction’s line pair can be resolved.

**Fig. 6:**
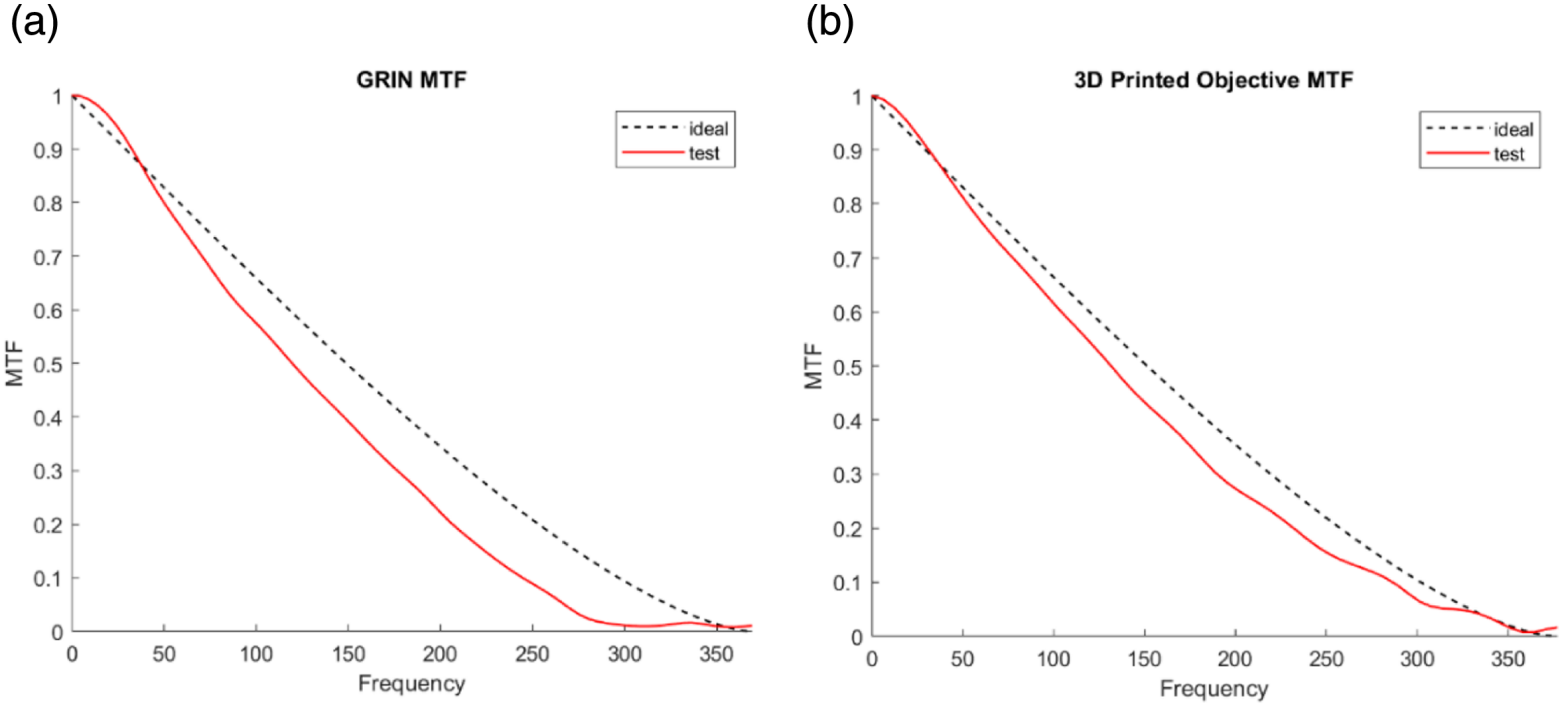
(a) Test on-axis MTF of the GRIN lens having a Strehl ratio of 0.82. (b) On-axis MTF of the 3D-printed objective having a Strehl ratio of 0.91 by the ISO 12233 slanted-edge method.

The uncoated objective and black-coated objective were tested with a negative 1951 USAF resolution target (R1DS1N1, Thorlabs Inc.) in Fig. 5. The working distance of the 3D-printed rod-like objectives was maintained at ∼1.6  mm, as specified in the design. As shown in Fig. 5, the contrast was significantly improved in the black-coated objective image in Fig. 5(b) compared with the image without coating in Fig. 5(a). The measured magnification was 4.13× with the system shown in Fig. 4, consistent with the expected 4× value based on the 1× miniature objective combined with 4× relay optics. The printed objective was able to resolve group 7 element 6 of the USAF resolution target, corresponding to a resolution of 4.38  μm, which closely matches the theoretical resolution of 4.27  μm calculated from the central wavelength of 525 nm defined by the filter used. To evaluate the performance of both the 3D-printed rod-like objective and the GRIN lens (model 1050-004597, Inscopix, Mountain View, California, United States) across the FOV, additional images were acquired at various lateral displacements. For the rod-like objective, as shown in Fig. 4(d), images were taken at five positions: −249  μm (left), −94.5  μm (L-in), 0  μm (center), +94.5  μm (R-in), and +249  μm (right). Group 7, element 6 remained resolvable at all these positions, demonstrating a total resolvable FOV of 498  μm (circled in yellow). For the GRIN lens, as shown in Fig. 4(e), images were acquired at −106.5  μm (L-out), −94.5  μm (L-in), 0  μm (center), +94.5  μm (R-in), and +106.5  μm (R-out), yielding a resolvable FOV of 188  μm (circled in green), beyond which group 7, element 6 was no longer resolvable (blue circle). Thus, the black-coated 3D-printed objective provided ∼7.0× resolvable area than that of the GRIN lens. Taken together, these results confirm that the 3D-printed rod-like objective—despite its smaller overall diameter—can resolve all features in group 7 comparably to the GRIN lens while providing a substantially larger FOV. In addition, the MTF is experimentally measured from slanted-edge images of a chrome on glass using an ISO 12233 implementation with software developed by Chidley et al.^21^ as shown in Fig. 6. This analysis is performed for both the commercial GRIN lens and the 3D-printed objective. Because the 3D-printed objective did not incorporate an aperture stop, the effective on-axis NA was 0.1004 and varied to an off-axis minimum NA of 0.075. On-axis, the measured Strehl ratio is 0.82 for the GRIN lens and 0.91 for the 3D-printed objective. To evaluate off-axis performance, the target is micro-positioned to the left and right edges of the 188-μm field. For the GRIN lens, a lateral shift of 94.5  μm yields an off-axis Strehl ratio of 0.36 (left) and 0.50 (right). In contrast, the 3D-printed objective maintains significantly higher off-axis performance: a shift of 94.5  μm produces a Strehl ratio of 0.87 (left) and 0.85 (right) as summarized in Table 2.

**Table 2:** On-axis and off-axis Strehl ratio for GRIN and 3D-printed objective.

	Left −94.5 μm	On-axis 0 μm	Right +94.5 μm
GRIN lens	0.36	0.82	0.50
3D-printed objective	0.87	0.91	0.85

**Fig. 7:**
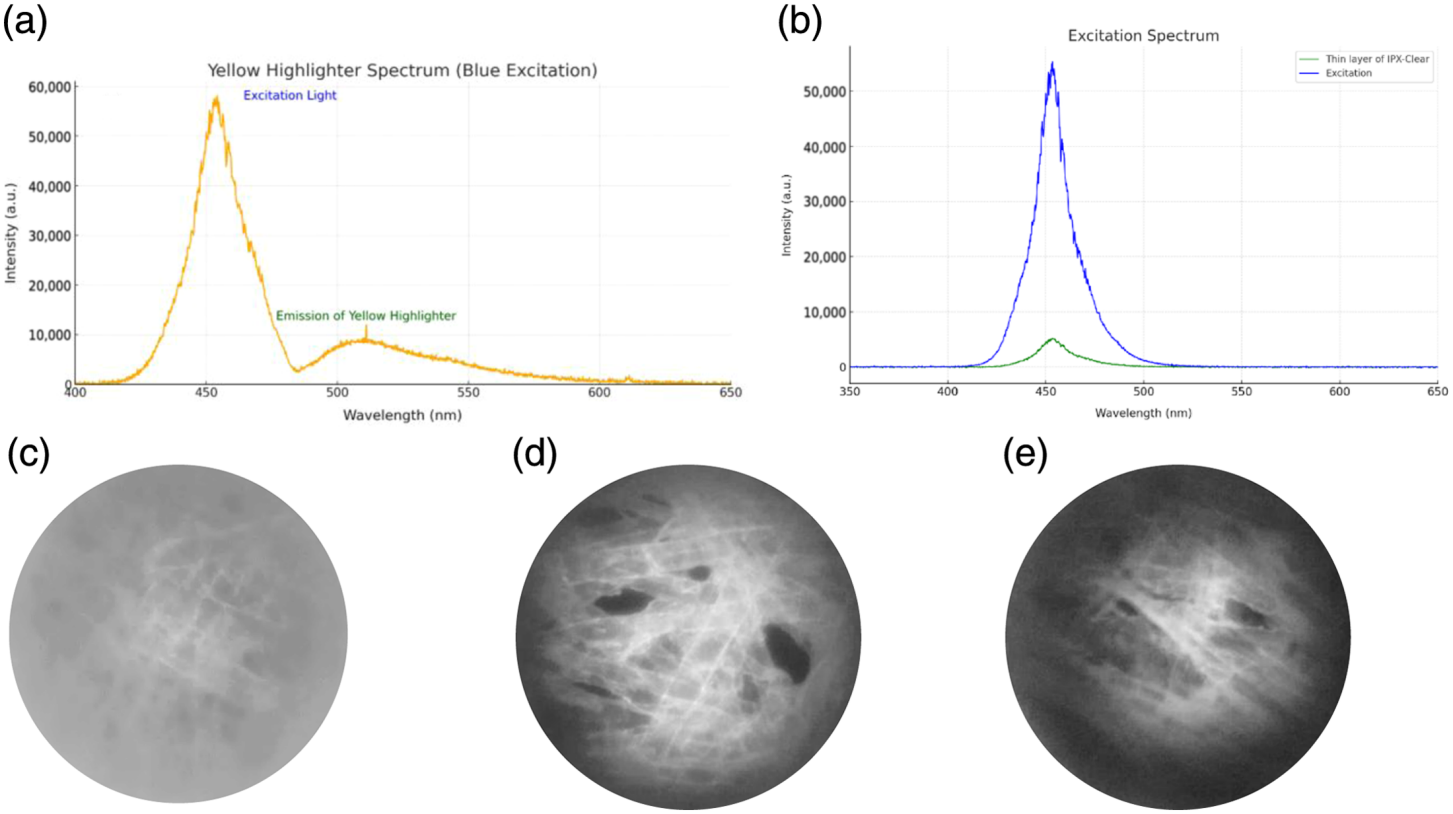
(a) Spectrum measured with an Ocean Optics spectrometer, where excitation light from the system passes through yellow-highlighted tissue paper, and the resulting fluorescence emission is captured. (b) Excitation spectra measured with an Ocean Optics spectrometer using a 100-ms integration time. The blue curve corresponds to the excitation light transmitted through a clean substrate, whereas the green curve corresponds to the same excitation light after passing through a substrate coated with a thin layer of IPX-Clear liquid. (c) Tissue paper highlighted in yellow imaged with the IP-S objective. (d) Tissue paper highlighted in yellow imaged with the IP-Visio objective. (e) Tissue paper highlighted in yellow imaged with the IPX-Clear objective.

A preliminary test was performed to assess contrast for fluorescence imaging using tissue paper (Fisherbrand lens paper, Thermo Fisher Scientific Inc., Waltham, Massachusetts, United States) stained with a yellow highlighter. The yellow highlighter was chosen because its emission peak closely matches that of proflavine (∼511  nm), as shown in Fig. 7(a), where the spectrum was measured using an Ocean Optics spectrometer. This yellow-painted tissue paper sample was imaged using IP-S, IP-Visio, and IPX-Clear printed lenses to obtain qualitative fluorescence images. As shown in Figs. 7(c)–7(e), the IP-Visio lens demonstrated significantly higher contrast in Fig. 7(d), confirming its reduced autofluorescence and suitability for fluorescence applications. In contrast, the IP-S 3D-printed rod-like objective produced the lowest image contrast among the three materials in Fig. 7(c), which aligned with previously observed high autofluorescence intensity in Figs. 3(a) and 3(b). Although the IPX-Clear 3D-printed rod-like objective produced high contrast features near the optical axis, darker peripheral regions were observed in Fig. 7(e). The darker edges observed in IPX-Clear are caused by absorption of the excitation light because off-axis rays pass through a different relative fraction of polymerized solid and unpolymerized resin than on-axis rays. Specifically, they pass through a greater proportion of unpolymerized resin toward the radial periphery. This hypothesis was verified by transmitting the 455-nm LED through a thin layer of liquid IPX-Clear resin and recording the spectrum with an Ocean Optics spectrometer as seen in Fig. 7(b). The measurement showed a significant reduction in transmitted intensity at the same integration time, confirming that the resin strongly absorbs excitation light. The darker peripheral region was attributed to the volume of the unpolymerized resin among the polymerized lenses. Light entering the off-axis regions of the objective experiences vignetting because more excitation light is absorbed by the unpolymerized resin. These results demonstrate that the excitation wavelength used in this testing system shown in Fig. 7(e) is not well-suited for structures containing unpolymerized IPX-Clear. Ultimately, IP-Visio was chosen as the material for 455-nm excitation wavelength in fluorescence imaging of tissue samples stained with proflavine.

**Fig. 8:**
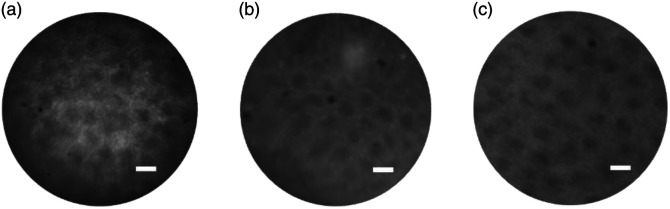
(a) Black-coated objective imaging colon tissue (scale bar=50  μm). (b) Commercial GRIN lens imaging the colon. (c) Reference image of the colon taken by 4× (0.13 NA) commercial objective without miniature objective (scale bar=50  μm).

This test verified that IP-Visio is suitable for this specific excitation wavelength with the current design where there is non-polymerized/unpolymerized resin in between the stacked lenses. For direct comparison, a colon tissue sample stained with proflavine was imaged using three lenses: the 3D-printed IP-Visio objective, a commercial GRIN lens, and a commercial 4× objective (Olympus UPlanFl), as shown in Fig. 8. Clear visualization of colonic crypts was achieved with both the 3D-printed IP-Visio objective and the GRIN lens, each providing ∼1× magnification. Compared with the GRIN lens, the 3D-printed objective captured a larger FOV image and showed the colonic crypts with higher contrast. The darker edge in the 3D-printed objective as shown Fig. 8(a) is attributed to a decrease in effective NA at larger field angles due to the absence of a physical stop, which reduces throughput at larger field angles.

## 4 Conclusion and Future Work

This work demonstrated the ability to fabricate a compact, rod-like objective composed of multiple convex refractive surfaces embedded in a lower-index material, all produced in a single print job using 2PP additive manufacturing. The convex surfaces were printed with fine 2GL parameters, whereas the outer wall was printed with coarser settings to reduce print time. The design featured an enclosed rod-like objective consisting of both polymerized and unpolymerized resin, eliminating the need for post-print alignment. To the best of our knowledge, this type of rod-like objective has not previously been reported.

The proof-of-concept lens maintained the compact form factor of a GRIN lens while functioning as a traditional objective, offering greater optical design freedom and improved correction. As no published data existed on autofluorescence across the full excitation range of 250 to 600 nm and emission range of 300 to 650 nm, we characterized IP-S, IP-Visio, and IPX-Clear in both liquid and cured forms using a full 3D EEM. Measurements with the Tecan Spark microplate reader revealed that, for fluorescence applications at excitation wavelengths between 450 and 575 nm, IP-Visio is the preferred material when uncured resin remains in the structure due to its low autofluorescence across the measured spectral range. IP-S exhibited high autofluorescence in the liquid state at 375 to 440 nm excitation and in the cured state at 450 to 550 nm excitation, making it unsuitable for fluorescence applications within this range. In contrast, IPX-Clear is the most optimal choice when the structure is fully polymerized.

The fabricated 500-μm-diameter rod-like objective resolved 4.38-μm features (group 7, element 6 of the USAF resolution target) across a resolvable span of 498  μm, closely matching expectations. In comparison, a 600-μm-diameter GRIN lens exhibited a much smaller resolvable span of 188  μm at similar magnification. This demonstrated that the 3D-printed objective provides a substantially larger resolvable imaging area while maintaining a smaller diameter. Apart from the high autofluorescence intensity observed in IPX-Clear liquid, tissue paper imaging revealed dark peripheral regions due to strong absorption from unpolymerized resin. Spectrometer measurements confirmed that 455-nm light was absorbed by thin layers of unpolymerized IPX-Clear, further validating that IP-Visio is the most suitable material for excitation at 455 nm. In conclusion, we demonstrated the fabrication of a proof-of-concept rod-like objective capable of resolving cellular features in the mouse colon. Future work will focus on integrating this objective with a fiber bundle for *in vivo* colon imaging. For fiber bundles which have core-to-core spacing of 3.3  μm (FIGH-30-650S, Fujikura Ltd., Tokyo, Japan), or 4.5  μm (FIGH-30-850N, Fujikura Ltd.), the magnification will need to be increased to ∼1.6× to 2.1× to match the fiber bundle sampling according to the Nyquist theorem. In addition, we will investigate modulating the laser power during printing to tune the refractive index, enabling the fabrication of a fully solid structure. As there is no physical stop placed in this miniature objective, a 3D-printed stop could be incorporated in the objective fabrication, and the 3D-printed rod-like objective can be expanded to non-axially symmetric designs.

## Supplementary Material

10.1117/1.JBO.31.3.036003.s01

## Data Availability

Data underlying the results presented in this paper are not publicly available at this time but may be obtained from the authors upon reasonable request.
