# Supplementary material for: Miniature 3D-printed rod-like refractive objective for endoscopic applications
Source: J Biomed Opt. 2026 Mar 10;31(3):036003. doi: 10.1117/1.JBO.31.3.036003 (PMC12975134; doi:10.1117/1.JBO.31.3.036003)
Supplement: Supplementary file 1 [file JBO_031_036003_SD001.pdf]

Supplementary information

IP-S Objective prescription

Radius (mm)	Thickness (mm)	Material	Clear Semi-Diameter (mm)	Conic
-	1.659	Air	-	0
-	0.500	IP-S solid (1.515)	-	0
-0.154	0	IP-S liquid (1.487)	0.235	-1.731
-	0.250	IP-S solid (1.515)	0.235	-
-0.228	0	IP-S liquid (1.487)	0.235	-1.189
-	0.280	IP-S solid (1.515)	0.235	0
-0.159	0	IP-S liquid (1.487)	0.235	-0.655
0.159	0.280	IP-S solid (1.515)	0.235	-0.646
-	0	IP-S liquid (1.487)	0.235	0
0.235	0.250	IP-S solid (1.515)	0.235	-1.187
-	0	IP-S liquid (1.487)	0.235	0
0.160	0.500	IP-S solid (1.515)	0.235	-1.813
-	1	Substrate (1.52)	0.235	0
-	0.982	Air	0.235	0

IP-Visio Objective prescription

Radius (mm)	Thickness (mm)	Material	Clear Semi-Diameter (mm)	Conic
-	1.659	Air	-	0
-	0.500	IP-Visio solid (1.514)	-	0
-0.135	0	IP-Visio liquid (1.489)	0.235	-1.643
-	0.250	IP-Visio solid (1.514)	0.235	-
-0.205	0	IP-S liquid (1.489)	0.235	-1.123
-	0.280	IP-S solid (1.514)	0.235	0
-0.148	0	IP-S liquid (1.489)	0.235	-0.663
0.148	0.280	IP-S solid (1.514)	0.235	-0.669
-	0	IP-S liquid (1.489)	0.235	0
0.214	0.250	IP-S solid (1.514)	0.235	-1.130
-	0	IP-S liquid (1.489)	0.235	0
0.146	0.500	IP-S solid (1.514)	0.235	-1.731
-	1	Substrate (1.52)	0.235	0
-	1.005	Air	0.235	0

IPX-Clear Objective prescription

Radius (mm)	Thickness (mm)	Material	Clear Semi-Diameter (mm)	Conic
-	1.659	Air	-	0
-	0.500	IP-Visio solid (1.547)	-	0
-0.090	0	IP-Visio liquid (1.526)	0.235	-1.263
-	0.250	IP-Visio solid (1.547)	0.235	-
-0.141	0	IP-S liquid (1.526)	0.235	-1.187
-	0.500	IP-S solid (1.547)	0.235	0
-0.125	0	IP-S liquid (1.526)	0.235	-0.739
0.128	0.500	IP-S solid (1.547)	0.235	-0.717
-	0	IP-S liquid (1.526)	0.235	0
0.166	0.250	IP-S solid (1.547)	0.235	-1.251

-	0	IP-S liquid (1.526)	0.235	0
0.108	0.500	IP-S solid (1.547)	0.235	-1.369
-	1	Substrate (1.52)	0.235	0
-	1.005	Air	0.235	0
